# Combining Aesthetic with Ecological Values for Landscape Sustainability

**DOI:** 10.1371/journal.pone.0102437

**Published:** 2014-07-22

**Authors:** Dewei Yang, Tao Luo, Tao Lin, Quanyi Qiu, Yunjian Luo

**Affiliations:** 1 Key Lab of Urban Environment and Health, Institute of Urban Environment, Chinese Academy of Sciences, Xiamen, China; 2 Xiamen Key Lab of Urban Metabolism, Xiamen, China; DOE Pacific Northwest National Laboratory, United States of America

## Abstract

Humans receive multiple benefits from various landscapes that foster ecological services and aesthetic attractiveness. In this study, a hybrid framework was proposed to evaluate ecological and aesthetic values of five landscape types in Houguanhu Region of central China. Data from the public aesthetic survey and professional ecological assessment were converted into a two-dimensional coordinate system and distribution maps of landscape values. Results showed that natural landscapes (i.e. water body and forest) contributed positively more to both aesthetic and ecological values than semi-natural and human-dominated landscapes (i.e. farmland and non-ecological land). The distribution maps of landscape values indicated that the aesthetic, ecological and integrated landscape values were significantly associated with landscape attributes and human activity intensity. To combine aesthetic preferences with ecological services, the methods (i.e. field survey, landscape value coefficients, normalized method, a two-dimensional coordinate system, and landscape value distribution maps) were employed in landscape assessment. Our results could facilitate to identify the underlying structure-function-value chain, and also improve the understanding of multiple functions in landscape planning. The situation context could also be emphasized to bring ecological and aesthetic goals into better alignment.

## Introduction

Various landscapes continually provide human society with multiple benefits, which are derived from ecological services and aesthetic attractiveness [1]–[5]. However, few researches gave consideration to aesthetic preferences in ecological assessment of landscapes [6], [7], and thus efforts towards landscape sustainability encounter the dilemma of making eco-aesthetically appealing landscapes [8]–[12].

Studies on landscape aesthetics have revealed two contrasting paradigms, which were either from an objective perspective (intrinsic attribute of the landscape) or a subjective point of view (human preferences) [1], [13], [14]. The employed methods varied from formalized indicators to aesthetic surveys [13], [15]. In the urban and regional planning, it still remains challengeable to construct eco-aesthetically appealing landscapes that meet multiple human needs [7], [10], [11], [16]–[19].

In attempt to combine aesthetic with ecological values, a number of models and/or indicators have been employed in landscape assessment [7], [20], [21]. Some studies have found that people appreciate aesthetically appealing landscapes that tend to be natural in ecological assessment [1]–[3], while some studies have indicated that ecologically sound landscapes may not be aesthetically pleasing [2], [22], [23]. This contradiction poses a challenge for efforts to combine aesthetic preferences and ecological service using a reliable assessment framework [7], [24], [25]. Moreover, the scale and spatial patterns of various landscape types need to be taken into account for both ecologically vital and aesthetically attractive.

Land use/cover change is a key socio-economic footprint of human activity and potentially affects aesthetic and ecological functions of landscapes [24], [26], [27]. The ecological services vary with different land-use types [28], [29], while the spatial patterns of land-use types give rise to various aesthetic experiences. Thus, to what extent do landscape types meet human needs in terms of ecological services and aesthetic attractiveness [3], [17], [23], [30], [31]? This would be useful to determine what measures of both ecological and aesthetic landscapes are potentially positive [20], [25].

Therefore, the aims of the present study included that: (1) a hybrid framework, which combine aesthetic and ecological landscape values, were proposed to assess integrated functional performances of different landscape types; (2) the framework was used to examine integrated functional performances and interrelationships of different landscape types in the Houguanhu Region of Wuhan City, central China; and (3) potential strategies for landscape planning were discussed. Our results could encourage an attempt of interdisciplinary research in a shared vision of landscape sustainability.

## Methodology

### Case study

The Houguanhu Region (113°41′–114°13′E, 30°15′–30°41′N) lies within the southwestern Wuhan city of Hubei Province in central China. Houguanhu Region covers an area of approximately 148.18 km^2^, and is mainly comprised of lakes and flatlands with a height of less than 100 m. The average annual temperature is 16.5°C and the total annual precipitation is 1,100–1,450 mm. Houguanhu Region has a population of approximately 63,300 people living in 78 settlements, and 79% of residents are classified as rural population. Regional planning for Houguanhu currently classifies it as an emerging “Ecological Livable Area” and “Tourist Resort”, and this region is experiencing rapid changes in landscape structures [5].

**Figure 1:**
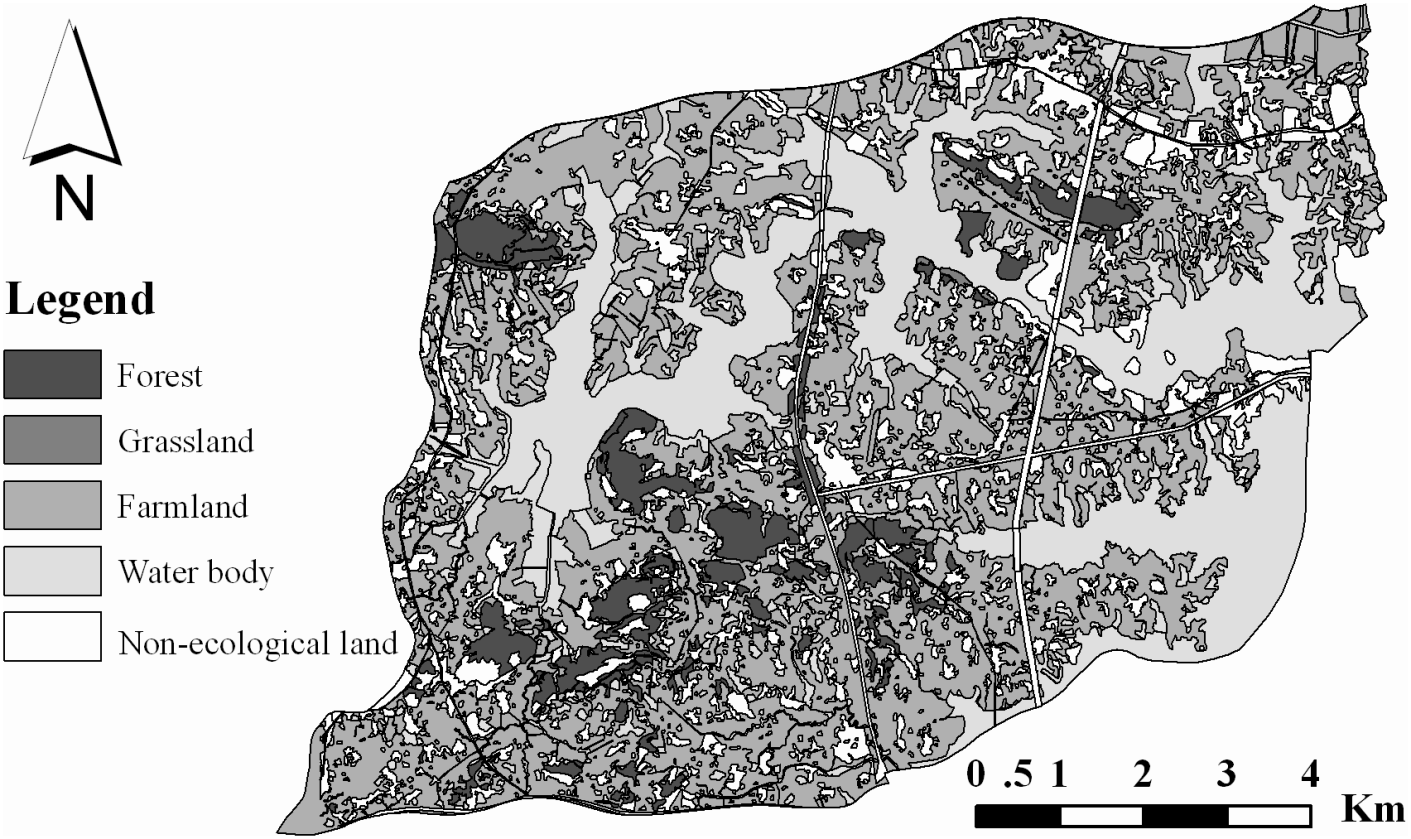
Landscape type map in Houguanhu Region. This Figure and following Figure 3a are redrawn according to Luo et al. [5] and Yang et al. [32].

The Houguanhu Region has a variety of diverse landscape resources and a rich historical culture. Five general landscape types, consisting of forest, farmland, grassland, water body and non-ecological land (i.e., desert in [28]), are shown in Figure 1, where the classification criteria follow Costanza et al. [28]. Non-ecological land includes rural settlement, built-up areas, roads, mines, industry zones and historic areas. The water body covers 30.56% of the total area of the Houguanhu Region, where the Zhiyin Lake and the Houguan Lake are the two largest lakes in this region. Farmland and scattered non-ecological land cover 46.75% and 15.98% of total area, respectively. Forest mainly in hilly areas and scattered grasslands occupy 0.21% of the total area. Forest and grasslands are mainly distributed in the south region, while farmland and non-ecological land are scattered across the entire area. The water body is mainly located in the central region.

In recent years, diverse landscapes have attracted a large number of tourists from outside the region. At the same time, however, rapid population growth and urban expansion have exerted intensive pressures on the natural environment and landscape. In response, since 2009 the local government has developed a series of regional landscape planning for sustainable development [5], [32]. Since cultural functions of landscapes usually receive less attention in landscape planning in China, this study may also benefit ongoing landscape planning in other similar regions.

### Field survey

The perception-based aesthetic landscape value evaluation method [1], [33] was employed in this study. A revised survey instrument of Von Haaren [33] was used to evaluate the landscape aesthetic value. The survey instrument consists of basic information, an aesthetic landscape value score and a viewshed map. Basic information (i.e. survey date, survey site coordinate, and site description) provided a landscape background for the 146 sample sites. The aesthetic landscape value score (*Vt = V1−V2+V3*) for each site included a positive score (*V1*), negative score (*V2*), and positive impact score of surrounding landscapes (*V3*). In each site, the biophysical landscape components (e.g., lakes, grassland, forest, hedgerows, electricity pylons, mines, and unsightly isolated buildings) were considered in the evaluation process. Landscape features (e.g., naturalness, openness, uniqueness, diversity, accessibility and visual comfort) also contributed to evaluation scores. The aesthetic landscape value was coded from 1 (lowest) to 5 (highest) on a 5-point rating scale. The viewshed map was sketched on each site for further revision using ArcGIS 9.2. Each viewshed map would be assigned an aesthetic landscape value score.

A total of 146 grid samples of 1 km^2^ were established within the study area using the uniform-grid-square sampling method. Basic maps (e.g., topographical map, landscape type map, administrative map) and socio-economic data (e.g., regional planning documents, and regional eco-economic statistic materials) were collected for spatial analysis using ArcGIS 9.2 tools.

The site survey of aesthetic landscape value was conducted from June 9th to 15th, 2010. Six surveyors from Institute of Urban Environment participated in a 3-hour field training program, and then conducted the formal survey as three two-person groups. During the survey, surveyors compared each other’s score and discussed until an agreement on a final score. After a 7-day survey, 141 valid viewshed maps with corresponding aesthetic scores were obtained. Blind viewshed areas without field aesthetic scores and five invalid survey samples were given aesthetic scores using spatial interpolation and joint method in ArcGIS 9.2. Consequently, a distribution map of aesthetic landscape value with 146 assigned samples was established. In this map, the study area was divided into graded aesthetic landscape value units based on a 5-point rating scale, with 5 being the highest value. The higher value will be more aesthetic attractive. Yang et al. [32] discussed in detail the survey instrument and data processes.

### Aesthetic landscape value coefficients

The landscape type map and the aesthetic landscape value distribution map were overlaid in ArcGIS 9.2 to identify the aesthetic value of different landscape types. Then the area of each landscape type was extracted for each graded aesthetic landscape value region. The aesthetic landscape value coefficient, which refers to the aesthetic landscape value of unit area per landscape type, was calculated as:
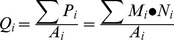
(1)Where *Q_i_* is the aesthetic landscape value coefficient (point/hm^2^) of the *i*th land type (forest, grassland, farmland, water body, and non-ecological land); *P_i_* is the aesthetic landscape value of the *i*th landscape types; *M_i_* is the aesthetic landscape value score of the *i*th landscape type; *N_i_* is the area of the *i*th landscape type in 5-grade aesthetic landscape value units; *A_i_* is total area of the *i*th landscape type.

### Ecological landscape value coefficients

According to the ecological service valuation framework of Costanza et al. [28], ecological service values of Chinese terrestrial ecosystems were evaluated by export judgment, which were developed by Xie et al. [29]. Ecological landscape value coefficients were calculated for five landscape types based on the sum of the eight ecosystem service values (see Table 1). The ecological landscape value coefficients were added up, where the recreation and cultural service were excluded, because they were inappropriate to simply add and subtract when comparing with monetary value. Based on the ecological value coefficients and the areas of the five landscape types, an ecological landscape value distribution map was calculated using Equation (2):
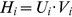
(2)Where *H_i_* is the ecological landscape value ($/hm^2^) of the *i*th landscape type (forest, grassland, farmland, water body and non-ecological land); *U_i_* is the ecological landscape value of the *i*th landscape type on a scale of 1–5; *V_i_* is the area of the *i*th landscape type.

**Table 1:** Ecosystem service values of Chinese terrestrial ecosystems (based on Xie et al. [29] and ecological landscape value coefficients of the five landscape types.

Ecosystemservices	Forest ($/hm^2^)	Grassland ($/hm^2^)	Farmland ($/hm^2^)	Water body ($/hm^2^)	Non-ecological land ($/hm^2^)
Atmospheric regulation	463.62	105.97	66.23	0.00	0.00
Climate regulation	357.65	117.12	115.81	59.85	0.00
Water conservation	423.88	104.10	78.07	2651.94	3.90
Soil formation and protection	516.60	253.75	189.99	1.30	2.60
Waste treatment	173.53	170.47	213.41	2365.68	1.30
Biodiversity conservation	431.83	141.84	92.38	324.01	44.24
Food production	13.25	39.04	130.13	13.01	1.29
Raw materials	344.40	6.50	13.01	1.29	0.00
Ecological landscape value coefficients	2724.76	938.80	899.04	5417.10	53.33

### Integrated evaluation of landscape values

The coefficients of ecological and aesthetic landscape value were normalized to evaluate integrated landscape values. The ecological and aesthetic landscape value coefficients of five landscape types were converted into values from −2 to 2 using Equation (3):
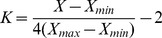
(3)Where *K* is the normalized aesthetic or normalized ecological landscape value coefficient of each landscape type; *X* is the aesthetic or ecological landscape value coefficient of each landscape type; *X_max_* and *X_mi_*_n_ are the maximum and minimum value of the aesthetic or ecological landscape value coefficient.

We assumed that the aesthetic and ecological landscape values contributed equally to the integrated landscape value. In order to quantify the functional roles of different landscape types in the integrated landscape values, the two normalized coefficients were visually displayed in a two-dimension coordinate system. The distribution maps of the aesthetic and ecological landscape value were overlapped into an integrated one of landscape value in ArcGIS 9.2, which were used to identify spatial characteristics of landscapes.

## Results

### Landscape value coefficients

Functional performances and value contribution of the five landscape types were quantified using a 5-point Likert Scale with 5 being the highest value. Aesthetic landscape value per unit area was in decreasing order: grassland, water body, forest, farmland, and non-ecological land (Table 2). The natural landscapes performed better than the artificial landscapes in terms of aesthetic functional performance.

**Table 2:** Aesthetic landscape value scores (P, point_*_hm^2^) of five landscape types.

Aesthetic landscapevalue units	Forest	Grassland	Farmland	Water body	Non-ecological land
1	0.00	0.00	34.32	4.80	20.82
2	0.00	0.00	313.94	112.48	160.72
3	144.76	0.82	2239.91	670.59	1350.75
4	2672.88	47.30	16046.76	8326.75	5069.18
5	1238.10	98.48	9884.85	10807.28	2743.78
Total	4055.74	146.60	28519.77	19921.89	9345.24
Aesthetic landscape value coefficients	4.21	4.61	4.12	4.40	3.95

The normalized aesthetic and ecological value coefficients were obtained using Equation (3) (Table 3). The aesthetic landscape value per unit area of water bodies was highest, followed by forest, grassland, farmland, and non-ecological land. Similarly to the aesthetic landscape values, natural landscapes had higher ecological values than cultivated or man-made landscapes.

**Table 3:** Normalized aesthetic and ecological landscape value coefficients.

	Forest	Grassland	Farmland	Water body	Non-ecological land
Ecological landscape value coefficients ($/hm^2^)	2724.76	938.80	899.04	5417.10	53.33
Normalized ecological value coefficients	−0.01	−1.34	−1.37	2.00	−2.00
Aesthetic landscape value coefficients (point/hm^2^)	4.21	4.61	4.12	4.40	3.95
Normalized aesthetic value coefficients	−0.44	2.00	−0.97	0.73	−2.00

### Combination of Landscape values

**Figure 2:**
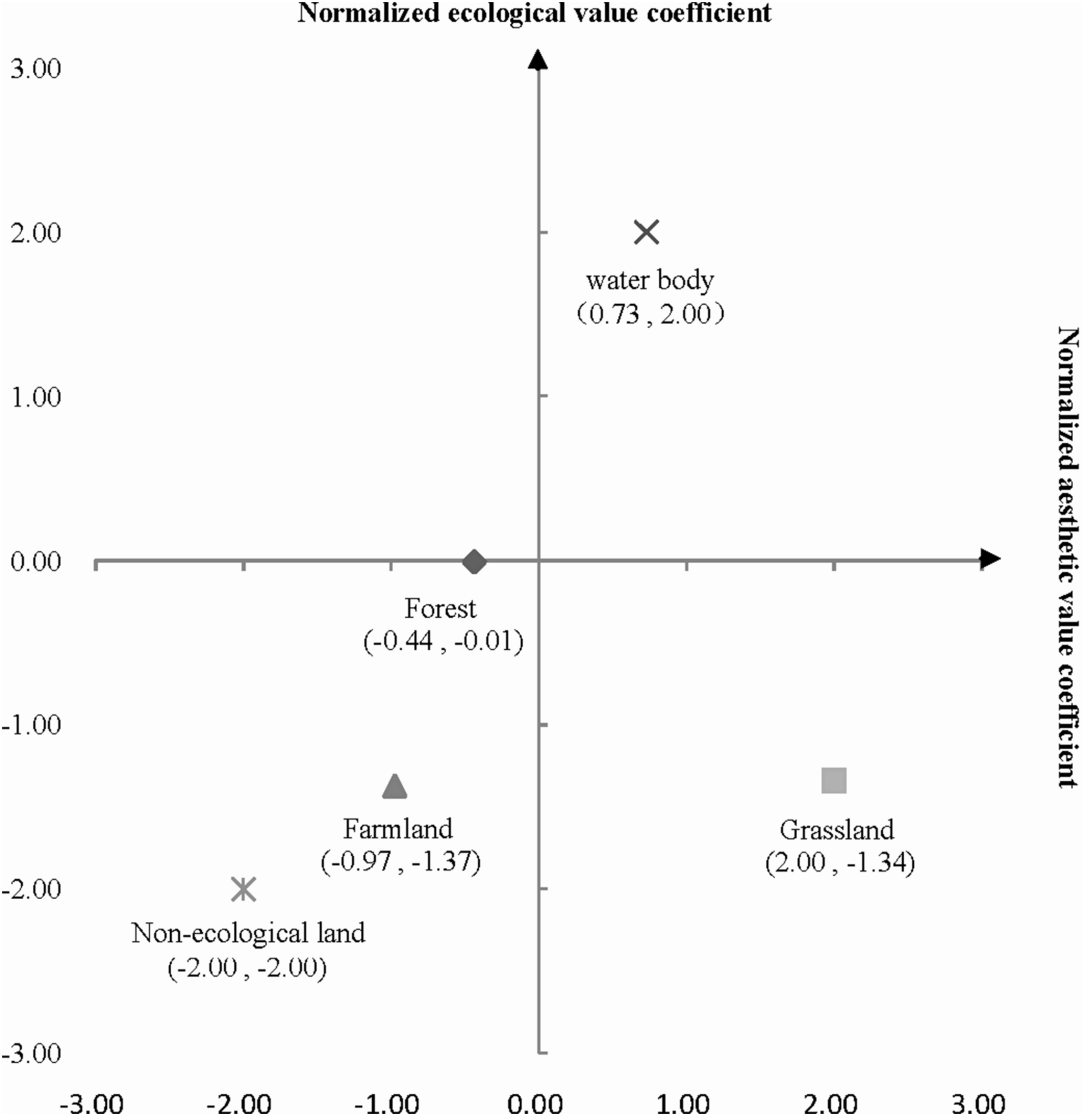
Two-dimensional coordinate system for landscape values.

We identified aesthetic, ecological and integrated functional performances of the five landscape types using a two-dimension coordinate system (Figure 2). In the two-dimension coordinate system, the five landscape types contributed unevenly, or even in opposite directions, to both landscape values. Water body was the only type where both landscape values were positive. Grassland was aesthetically attractive, but it was not ecologically valuable. The other three landscape types (i.e. forest, farmland, and non-ecological land) showed a positive relationship on aesthetic and ecological landscape value, respectively, but made a negative contribution to aesthetic and ecological landscape value.

### Distribution of landscape values

**Figure 3:**
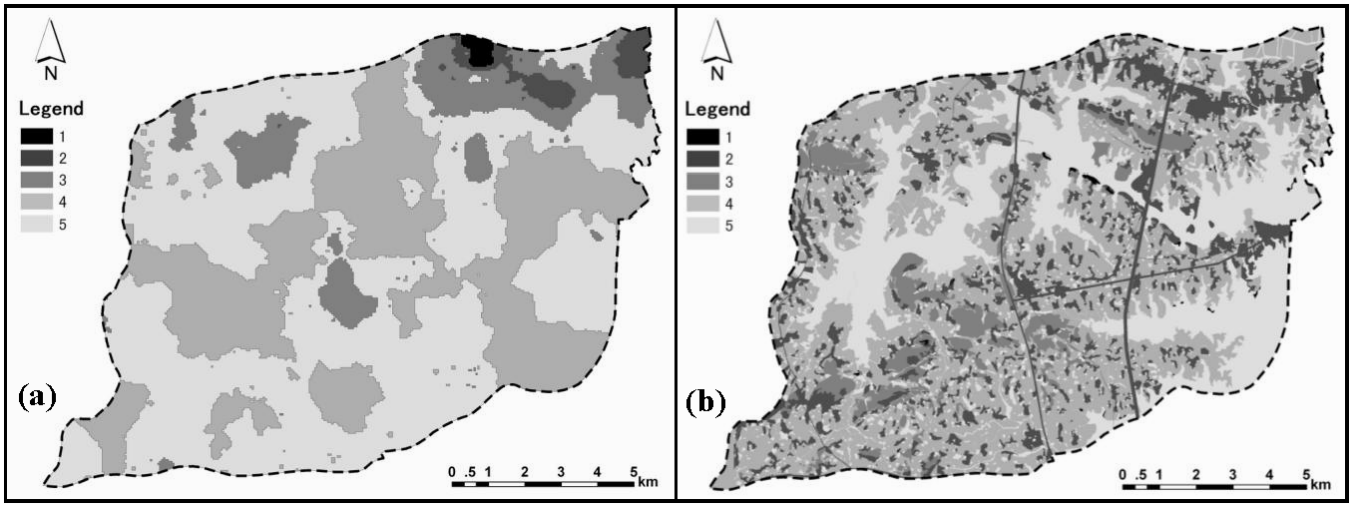
(a) Aesthetic landscape value distribution map and (b) ecological landscape value distribution map in Houguanhu Region.

**Figure 4:**
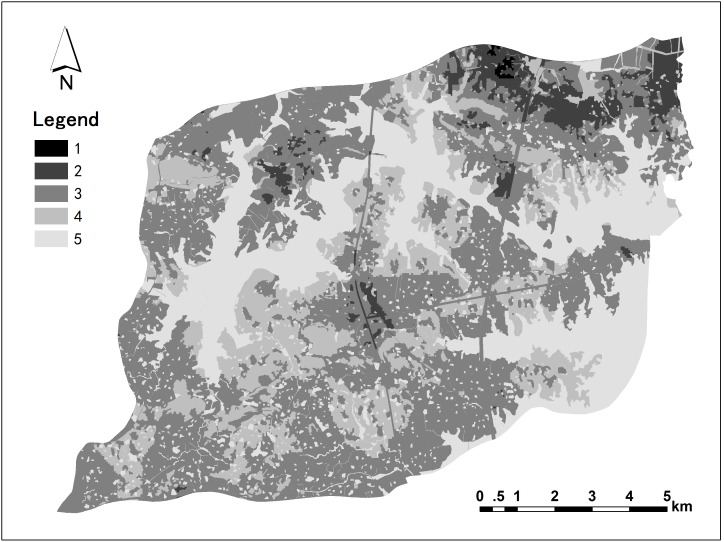
Integrated landscape value distribution map.

We drew the aesthetic landscape value distribution map after the aesthetic landscape survey (Figure 3a), and the ecological landscape value distribution map based on Equation (2) (Figure 3b). The integrated landscape value distribution map was obtained from the overlay of the aesthetic and ecological landscape value distribution maps in ArcGIS 9.2 (Figure 4). The landscape value scores ranged from 1 (lowest) to 5 (highest).

The aesthetic, ecological and integrated landscape values varied with the human activity intensity, and gradually decreased from the external water body to the transitional farmland and central non-ecological land. Central water body and adjacent areas received the highest values, the lowest was in northeastern built-up areas, and the moderate scores were in most other areas.

The distribution of aesthetic landscape value showed a large-scale spatial continuity, with the exception of the northeastern region. The central water body with visual characteristics of broad sight, good order and naturalness obtained higher aesthetic landscape value scores, while scattered non-ecological land and farmland with disorderliness, high-intensity buildings and fragmentation received lower evaluation scores. In contrast, the distribution of ecological landscape values exhibited a mosaic distribution with an unclear spatial trend. However, water body and their adjacent areas received higher ecological value scores.

## Discussion

### Metrics for evaluating landscape values

The functional performances and interrelationships between the five landscape types were depicted as the integrated landscape value in a two-dimension coordinate system and displayed in landscape value distribution maps. In contrast to objective aesthetic approaches [3], [13], [24], [34], the subjective landscape aesthetics in this study reflects the intrinsic perception of the forms and spatial configuration of landscape elements. Compared with conceptual models [7], [35], [36], our metrics (i.e., survey, coefficients, the two-dimension coordinate system and scopes) may quantitatively capture the functional performance of different landscape types.

### Landscape functions/values and landscape attributes

Our results showed that landscape values were strongly related to landscape attributes and the human activity intensity. Unlike semi-natural and human-induced landscapes, such as farmland and non-ecological land, the natural landscapes (i.e., water bodies and forest) contributed positively to both aesthetic and ecological functions. The controversial relationships (i.e., coincidence or disjuncture) between aesthetic performances and ecological quality have raised wide concerns [2], [7], [8], [20], [37]. These relationships can be quantified from the two-dimension coordinate system and distribution maps in this study, e.g., the negative or positive contribution level of landscape types in landscape values.

Human activities can result in the decrease of landscape values by changing natural landscapes into semi-natural and artificial landscapes. It was found that the landscape values increase from the human-dominated and semi-natural landscapes to more natural landscapes. The underlying structure-function-value chain can be induced from the modified landscape gradient influenced by human activities, which has been discussed in some landscape assessments [6], [36], [38], [39].

### Implications for landscape planning

The functional performances and distribution of landscape values were identified in present study, which may help optimize landscape planning in terms of developing priorities and intensity. The two-dimension coordinate system indicated that replacing one landscape type with another may result in a positive or negative change in the comprehensive landscape value. For example, more value would decrease if the water body was replaced by the non-ecological land than by the farmland. Thus, in order to bring aesthetic needs and ecological goals into better alignment, a functional compromise may encounter in landscape planning. In the landscape value distribution maps, the gradients of inherent landscape values and the concerns of underlying landscape value can be used to identify appropriate strategies, such as protection, restoration, and reconstruction. The protection and restoration measures may be taken to expand natural landscape spaces, while human-influenced landscapes should be reconstructed in an orderly way. The reconstruction measures (e.g., building hedgerow buffer zones, reducing the cutting effect of a road network on the overall landscape, or increasing accessibility between landscapes) may be involve in landscape planning.

The situational context (e.g. the social phase, indigenous culture and stakeholder demands) should also be incorporated into landscape planning in response to various concerns regarding the landscape functions [2], [24], [40]. Tourists are likely to care more about farmland aesthetics, while farmers put a higher value on agricultural productivity. However, both farmland attractiveness enjoyed by tourists and production features valued by farmers are likely to undermine ecological functions. Therefore, it was recommended for policy makers to align aesthetic features to better support ecological health. It was also recommended that the disorderly non-scenic regions which were important to the overall landscape (e.g. rural settlements, roads, and historic sites) should be carefully enhanced using green corridors and an ordered appearance to foster a more positive aesthetic experience.

### Limitations and future improvements

Some limitations and possible improvements of this study included:

1. In order to make the survey easier, site aesthetic evaluation was kept simple, and only five major landscape types were identified. It is recommended that the subdivisions of landscapes should be adopted for future research to convey more detailed information. In addition, surveyors from a variety of cultural backgrounds could be involved to incorporate a greater diversity of perspectives into landscape planning.
2. More comprehensive aspects of cultural landscape values (e.g. knowledge systems, social relations, and aesthetic values) could be incorporated into future studies to address the overall performance of landscape resources in the decision-makings.
3. Subjective aesthetic landscape coefficients can only convey information related to the relative importance of landscape values of the five landscape types. This may limit the use of aesthetic landscape coefficients when comparing with ecological evaluations.

## Conclusions

Due to the difficulties in integrating different data types, quantitative models and interdisciplinary studies, it was a far-reaching challenge to connect subjective (aesthetic) and objective (ecological) aspects in an integrated landscape assessment. Nevertheless, the landscape sustainability requires a careful investigation of the relationship between ecology and aesthetics. Compared to conventional conceptual frameworks, the metrics (i.e. field survey, normalized landscape value coefficients, and a two-dimensional coordinate system) were employed in this study in order to quantify the functional performances of landscape types and to present an integrated landscape value. The results bring aesthetic and ecological goals into a better alignment in landscape planning. The interdisciplinary approach may lead to a revolution in landscape designing and planning, because it balances ecological and aesthetic functions for landscape sustainability.
